# Blind Recognition of Forward Error Correction Codes Based on Recurrent Neural Network

**DOI:** 10.3390/s21113884

**Published:** 2021-06-04

**Authors:** Fan Mei, Hong Chen, Yingke Lei

**Affiliations:** School of Electronic Countermeasures, National University of Defense Technology, Hefei 230000, China; meifan14@nudt.edu.cn (F.M.); chenyue@nudt.edu.cn (H.C.)

**Keywords:** recurrent neural network, forward error correction codes, blind recognition, non-cooperative system, parameter initialization

## Abstract

Forward error correction coding is the most common way of channel coding and the key point of error correction coding. Therefore, the recognition of which coding type is an important issue in non-cooperative communication. At present, the recognition of FEC codes is mainly concentrated in the field of semi-blind identification with known types of codes. However, the receiver cannot know the types of channel coding previously in non-cooperative systems such as cognitive radio and remote sensing of communication. Therefore, it is important to recognize the error-correcting encoding type with no prior information. In the paper, we come up with a neoteric method to identify the types of FEC codes based on Recurrent Neural Network (RNN) under the condition of non-cooperative communication. The algorithm classifies the input data into Bose-Chaudhuri-Hocquenghem (BCH) codes, Low-density Parity-check (LDPC) codes, Turbo codes and convolutional codes. So as to train the RNN model with better performance, the weight initialization method is optimized and the network performance is improved. The experimental result indicates that the average recognition rate of this model is 99% when the signal-to-noise ratio (SNR) ranges from 0 dB to 10 dB, which is in line with the requirements of engineering practice under the condition of non-cooperative communication. Moreover, the comparison of different parameters and models show the effectiveness and practicability of the algorithm proposed.

## 1. Introduction

FEC coding is the most common way of channel coding and the key point of error correction coding. whose characteristics of mathematics ensure the receivers to correct errors that occurred during the process of transmission, it improves the quality of communication systems and has good real-time performance. The FEC coding types are widely used in non-cooperative communication fields such as adaptive modulation coding (AMC), cognitive radio and remote sensing communication [1]. In the field of information countermeasure, the type of error correction codes of the detected link must be recognized correctly, so as to obtain effective information payload and finally decipher the message of the sender [2]. However, in the context of non-cooperative communication, there is no prior knowledge of the FEC coding types adopted on the detection link, Therefore, it must be identified by the corresponding data analysis methods. In addition, this is the core content of the paper—the blind identification of FEC coding types. Blind recognition of error-correcting code types is also of great significance to improve the accuracy and efficiency of code parameter recognition.

At present, the recognition of FEC coding types can be divided into two types: full blind recognition and half blind recognition. The full blind recognition is the forward error correction coding type recognition analysis under the condition that the access signal information is completely unknown. However, in some special cases, it is possible to obtain a set of forward error correction coding types that the sender may adopt through some prior knowledge. Then, using the intercepted data flow, the forward error correction coding method with the largest matching degree is searched from the alternative set as the estimation of the coding types used in the detection link, which is the basic feature of semi-blind recognition.

At present, there is extensive research on parameter estimation technologies such as linear block codes [3,4,5], convolutional codes [6,7,8], LDPC codes [9,10], BCH codes [11] and Turbo codes [12,13]. However, these algorithms are based on known coding type, solving its generation matrix and generation polynomial to realize parameter identification. Under the condition of non-cooperative communication, the unknown encoding type of receiver will make it hard to the recognition of specific parameters. Therefore, it is crucial to study the recognition of types. At present, the recognition of FEC encoding types is mainly accomplished by mathematical methods according to the large amount of data received. In [14], the author proposed a type recognition algorithm based on rank criterion by using rank characteristics of receiving code words. However, the algorithm only analyzed the rank characteristics of each type separately and didn’t comprehensively analyze and identify them. In [15], a type recognition algorithm for linear block codes and convolutional codes based on run-length features was proposed. This algorithm could recognize linear block codes and convolutional codes well, but it failed to recognize other encoding types. In [16], a coding system identification strategy for error-correcting codes with unknown coding sequences was proposed, which could provide a basic identification process for type recognition. However, it did not study and analyze the coding type identification under the condition of bit error and failed to meet the basic requirements of engineering practice. In [17], the author proposed a general linear block code recognition and analysis method, which had certain engineering practicability and succeeded in improving the recognition efficiency to a certain extent. Despite these considerable advantages, the algorithm lacked a strict mathematical proof. At the same time, this method was also semi-blind and was hard to be used under the condition of full blind recognition. However, the recognition accuracy fell too fast in low SNR environment, failed to meet requirement in practice. Hence, a new identification algorithm with low complexity and high fault-tolerance is needed, which will be applied to many types of forward error correction codes and is convenient for engineering and practical application.

Deep learning is a subfield of machine learning. Traditional classifiers include linear discriminant analysis (LDA) [18], multi-level perception (MLP) [19] and support vector machine (SVM) [20]. As an emerging method, it has had a transformative impact on the development of natural language processing [21], computer vision [22] and other fields. Deep learning extracts features through multi-layer nonlinear compound operations, which also has a good data expression effect for complex data distribution. In terms of adaptive modulation and coding, it has also achieved satisfactory results [23,24,25,26]. The core of deep learning method is hierarchical representation learning, which aims to extract more complex high-level features by combining low-level features, so as to solve the previous tough problems requiring manual design of features [27]. It requires a great number of sample data for training the obtained nonlinear function [28]. The classification accuracy of the function for unknown data is significant. So deep learning can be used to deal with many kinds of signal processing problems. In recent years, deep learning has been draw into the area of recognition of code types. In [29], the authors constructed a deep fusion model based on 1-D convolutional layer and modified 1-D inception architecture to recognize the types of FEC codes and in [30], the authors proposed a convolutional neural network (CNN) model improved by embedding and block mechanism to classify the FEC codes. It achieves higher recognition performance than the algorithms which are based on traditional deep learning. However, these algorithms succeed to recognize the code types in 10 dB or even higher SNR and fail to achieve the goal in low signal to noise ratio.

In the paper, a code type recognition method based on RNN is raised. The novel method can identify the type of code through the classification process under low signal-to-noise ratio conditions. Furthermore, the advantage of this method lies in that any prior knowledge of encoder algorithm is not needed. Moreover, unlike traditional feature extraction methods, this method can automatically extract high-level features and improve the performance of FEC code type recognition. At the same time, compared with typical CNN, Simple RNN further considers the internal feature relationship of the code word, which improves the recognition accuracy. So as to improve the performance of Simple RNN furtherly, we come up with a weight initialization method. The results indicate that the improved RNN precedes Simple RNN or a single inception network. The major contributions of the paper are summed up as follows.

1. Aiming at FEC codes in non-cooperative communication system, a blind type recognition method based on RNN is proposed.
2. We adapted a new weight initialization method in RNN network to improve training efficiency and training accuracy and achieved end-to-end extraction of features. The gradient disappearance is effectively suppressed, the computational complexity is reduced and the training efficiency is improved.
3. We constructed a comprehensive and in-depth study on the performance of FEC coding type recognition on account of recurrent neural network. Experimental results indicate that compared with traditional networks and other single neural networks under low signal-to-noise ratio conditions, the performance of the Improved RNN is remarkably improved.

## 2. The Recognition Process

**Figure 1:**
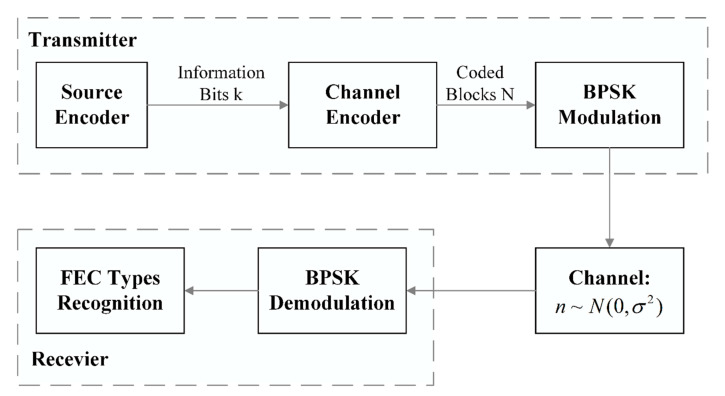
General block diagram of the blind recognition.

The section presents the schematic diagram of the digital communication system based on FEC coding recognition, as shown in Figure 1. It includes source coding, channel coding, modulator, demodulator, data storage and abundant channel simulation modules which aims to put the simulated channel propagation model into use of simulated code data.

Generally, source coding technique needs to compress data in order to transmit information more quickly, while channel coding technique is on the contrary. So as to assure the reliability of transmission, redundant codes, called supervised codes, will be added to the information codes to form a certain constraint relationship. We assume that bits of information from transmission is encoded to obtain the code words of code length N(N>k) through channel coding at the transmitter end and then mapped to the symbol vector through BPSK modulation. In the model, the channel adopts additive white Gaussian noise channel (AWGN) and the Gaussian noise samples are distributed to $n\sim N(0,\sigma^{2})，\sigma \in R$. The SNR is defined as the bit error rate (BER) and bit energy noise ratio (Eb/N0).

The received codeword is defined as r, which is also called the receiving codeword. Because the symbol vector is disturbed by noise through AWGN channel, then is defined as:

(1) r=e+v

where e is the error pattern. In general, BPSK demodulation is performed before the types of forward error correction codes are identified. On the basis of the Gaussian distribution of channel noise, the logarithmic likelihood ratio (LLR) of the transmitted information is showed as:

(2) $$LLR(r)=\ln\frac{P(v=0|r)}{P(v=1|r)}=\frac{2}{\sigma^{2}}$$

σ is the variance of the channel noise and r is the code words of reception and the recognition model of FEC code is used to identify the information bit vector sent by r. In this work, we focus on the recognition of types of FEC codes.

## 3. The Proposed Approach

In the section, we expound in detail the identification method of FEC coding type based on RNN and we will apply and verify the reliability of this method in the next section.

### 3.1. Data Generation

In the experiment, we use MATLAB to generate data sets of four different coding types. It is assumed that the information in receiving end has been successfully demodulated and perfect frame synchronization has been achieved. Hence, we consider using the BPSK modulation stream at the receiver of the AWGN channel. In the paper, four kinds of FEC codes are used, including LDPC codes and Turbo codes which can be close to the channel capacity. The LDPC codes is a kind of common linear block code, it is characterized by the sparsity of the test matrix of LDPC codes. LDPC code is a block code whose checksum matrix contains only a small number of non-zero elements. It is the sparsity of the check matrix that ensures both the decoding complexity and the minimum code distance increase linearly with the code length. The code itself is indistinguishable from any other block code except that the check matrix is sparse. Turbo codes, also known as cascaded convolutional codes, are based on the combination of recursive system convolutional codes and interleaved techniques. In the paper, the Turbo encoder is set to a fixed size and the initial states of the Recursive System Convolution (RSC) encoder are all zeros. In addition, these two codes, we also recognize BCH code and convolutional code. The BCH codes is a kind of linear block codes, it has strong error-correcting ability, convenient construction, simple coding and decoding and its performance is close to the theoretical value under short and medium code length. Compared with the linear block codes, the convolutional codes are different in that the codes of the current moment are not only related to the information elements of the current moment, but also related to the input information elements of previous moments. The correlation between the code sets makes the convolutional codes have better performance under the condition of the same bit rate and device complexity.

The work in the article covers the most common codes in the literature, including the following code types:

Block codes: BCH codes, Turbo codes, LDPC codes.

Convolutional codes: 1 bit input, 2 bits output and constraint length M = 5 to 8.

The block codes are usually expressed as the form (n,k), where k represents the information bits, n represents the total length of the block codes and r=n−k represents the length of the monitor bits. Under the condition of GF(2), a group of block codes C can generate a total $2^{k}$ of different code words. The expression is:

(3) $$C=(c_{0},c_{0},\ldots ,c_{k-1})$$

In this paper, we use the coding structure and parameters recommended by the consultative committee for space data systems (CCSDS)standard to generate the training and test data sets of LDPC codes and Turbo codes. LDPC codes set consists of three code parameters defined in [31], block length 1024, rate R = 1/2, 2/3, 4/5. The Turbo codes set consists of four parameters defined in block length 1784 and bit rate R = 1/2, 1/3, 1/4 and 1/6. BCH selects four code words, which are B (6,3), B (7,4), B (12,8) and B (15,11). The parity matrix is taken from [32].

In this paper, $\left(n,k,N_{0}\right)$ is used to describe the convolutional code, where k is the number of bits at the input end, n is the number of bits at the output end and $N_{0}$ is the length of the coding constraint. Standard rate 1/2 convolutional codes and different constraint lengths are considered as candidate codes, with constraint lengths ranging from 5 to 8. The octal representation of the different code generation polynomials is shown in Table 1.

### 3.2. Proposed Deep Neural Network Methodology

#### 3.2.1. Introduction of Simple RNN

In recent years, neural network is a hot research object. Further research on artificial neural network promotes the development of deep learning. At present, deep learning has made a series of remarkable achievements in image, text, video, Automatic Speech Recognition (ASR) and other application fields. RNN is a typical network structure in the field of deep learning. Its characteristic is that it can model the temporal changes of sequence data and is suitable for processing it. Because of this characteristic, RNN has been successfully applied in language modeling, speech recognition, sentiment analysis, machine translation and many other aspects and is expected to show a better application prospect in these aspects. Compared with CNN, the innovation of RNN lies in that by introducing the processing of the state of the last time step, the network can make full use of the information of the previous state in the current time and can model the change of sequence data in time, which is suitable for processing the data that changes in time dimension.

RNN includes input layer, hidden layer and output layer. In addition to fully connection between layers, the nodes of the hidden layer are connected to each other. However, there is no correlation between the nodes of each layer. Through such a design, the state of the hidden layer at the last moment can be added to the calculation of the hidden layer and the output layer at the current time, which is equivalent to using the previous state information to a certain extent. Theoretically, speaking, the current input of the hidden layer can not only be limited to the output of the previous stage of it, but also be related to the hidden layer outputs of the previous several time slices. However, in practice, in order to reduce the complexity of network structure, it is usually assumed that the output of the current hidden layer is only related to the output of the previous stage.

**Figure 2:**
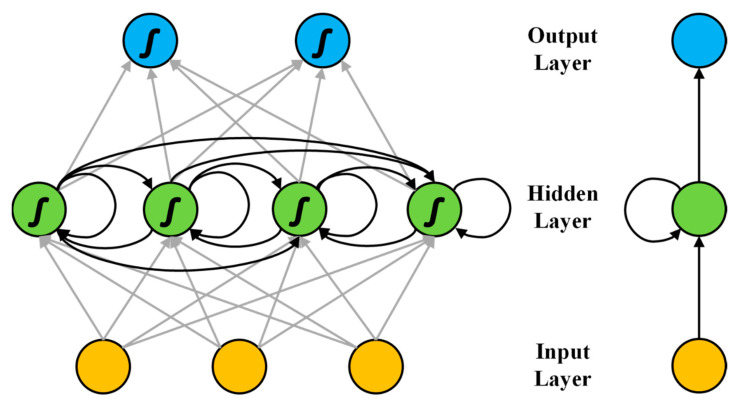
The basic model of simple Recurrent Neural Network.

Simple RNN is a typical structure and Figure 2 shows the basic model of it.

As the figure shows, the hidden layer of Simple RNN is the key of its structural design. In addition, to passing from the input layer to the hidden layer and from the hidden layer to the output layer, the hidden layer is also related. For Simple RNN, the forward propagation process is as follows:

(4) $$a_{h}^{t}=\sum_{i=1}^{I}w_{ih}x_{i}^{t}+\sum_{i=1}^{I}w_{h^{\prime }h}b_{h^{\prime }}^{t-1}$$

(5) $$b_{h}^{t}=\theta_{h}(a_{h}^{t})$$

(6) $$a_{k}^{t}=\sum_{i=1}^{H}w_{hk}b_{h}^{t}$$

$a_{h}^{t}$ represents the calculation output of the hidden layer and $a_{k}^{t}$ represents the output of the output layer. As the Equation (4) shows, the calculation of $a_{h}^{t}$ is divided into two parts. The first part is both connected to the input layer and calculated. The second part is the self-connecting of the hidden layer, that is, the output of the hidden layer from the last time step after passing the activation function, which $w_{h^{\prime }h}$ represents the weight parameters between the self-connecting of the hidden layer. $\theta_{h}$ represents the activation function through which the hidden layer output passes. The training optimization of Simple RNN is also optimized by BP algorithm. However, Simple RNN is propagating forward, it takes advantage of the timeline information. Therefore, the back propagation algorithm of Simple RNN transfers residuals back to the last time from the next time and the time dimension is equivalent to the number of layers of the deep neural network. Despite these outstanding advantages, the convergence speed of Simple RNN is slow in the training process and there is still a lot of room for improvement in the training speed.

#### 3.2.2. Weight Initialization of Simple RNN

Parameter initialization is the key part of neural networks. Excellent initialization can not only greatly improve the training efficiency, but also greatly improve the final training effect. At present, the weight initialization methods commonly used in neural networks are as follows:

1. Gauss initialization method initializes the weight to a random number that conforms to a Gaussian distribution with a mean of 0 and a standard deviation of 0.01. This is by far the most widely used initialization method in deep learning.
2. Xavier initialization method is to randomly initialize weights from a Gaussian or standard distribution multiplied by a certain ratio.
3. MSRA initialization method is to initialize to a Gaussian distribution with a mean of 0 and a variance of $\frac{2}{n}$.

In this section, we devote to solve the gradient explosion and gradient convergence problems without deeply changing the network structure of Simple RNN through weight initialization work and at the same time maintain long-term memory and have the ability to learn long time series. In this paper, a RNN weight initialization method proposed by Xu Xu et al. [32] is adopted to improve the performance of Simple RNN.

Gaussian initialization is adopted in this algorithm. For the self-connect weight of the hidden layer, initialize it here with the matrix W shown below.

(7) $$\begin{array}{ccccc} 1+\theta & \theta & \ldots & \ldots & \theta \\ \theta & 1+\theta & \theta & \ldots & \theta \\ \theta & \ldots & 1+\theta & \ldots & \theta \\ \theta & \ldots & \ldots & 1+\theta & \theta \\ \theta & \ldots & \ldots & \theta & 1+\theta \end{array}$$

As shown in Equation (7), the matrix used can be regarded as adding an identity matrix on the basis of a Gaussian initialization:

(8) W=I+A

**Figure 3:**
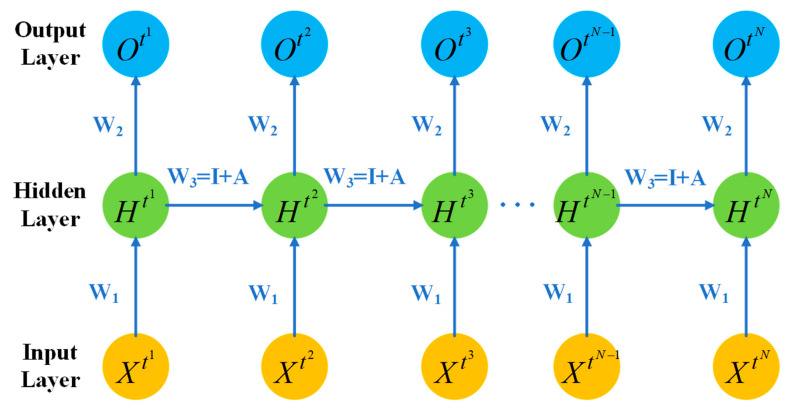
The weight initialization of Simple RNN.

I is the identity matrix, A is the Gaussian distribution matrix. θ is a Gaussian distribution with a mean of 0 and a variance of 0.01. The above initialization method for Simple RNN is shown in the Figure 3.

As shown in the Figure 3, X represents the input of Simple RNN at different time steps, H represents the state of the hidden layer at different time steps and O represents the state of the output layer of network at different time steps. In the ordinary neural network, the parameters are different between each layer. The parameters of each layer are the same for different time steps, since they are unrolled along the timeline. Therefore, for different time steps, the weight from the input layer to the hidden layer obtains $W_{1}$, the weight from the hidden layer to the output layer is always $W_{2}$ and the weight of the self-connecting of the hidden layer is always $W_{3}$. The weight of $W_{3}$ is initialized to I+A. $W_{1}$ and $W_{2}$ are initialized with the Gaussian distribution mentioned above. According to Equation (4), when the residual is calculated and it returned from the hidden layer is received by a time step, the residual of the next time will be multiplied by the weight of the hidden layer. This internal weights of hidden layer neurons are initialized as the matrix I+A which is described above, on the premise of assuming no other residual accumulation, as the hidden layer of residual travels forward by back propagation algorithm and is multiplied by unit matrix I, the next time the hidden layer of residual will remain the same and is multiplied by matrix A which is in line with gaussian distribution. The residual is multiplied by a certain coefficient to control the influence of the previous time step state on this time step. To a certain extent, the improved Simple RNN has the ability to learn long time series correlation, which can have better performance. Compared with the original, the improved Simple RNN has enhanced performance, effectively suppress the gradient disappearance, reduce the computational complexity and improve the training efficiency.

## 4. Simulation Results

In this section, the recognition of the four FEC codes based on the improved RNN network is first simulated. Further considering that the performance of neural network identification is affected by many factors, such as network type, data size, etc., this paper compares different network types, data sizes and the lengths of data, makes comprehensive comparison and summarizes in the end.

### 4.1. FEC Code Data Test Environment

The experimental setup simulates generating 1 million samples, each of which is 10,000 in length and stored in int32. The SNR of the sample is evenly distributed between −10 dB and 10 dB. The data set containing 70% of the samples was used as the training channel coding type recognition model and the data set containing 30% of the samples was used as the performance evaluation of the model. All training and testing are done in Keras using Nvidia 1080 Ti GPU. In the training process, we set learning rate as 0.002, if training loss has not reduced after every 10 epochs, set the study rate as 10% of the primary. Training network using batch size as 16 to train 200 epochs.

### 4.2. Recognition Performance of Code Type

**Figure 4:**
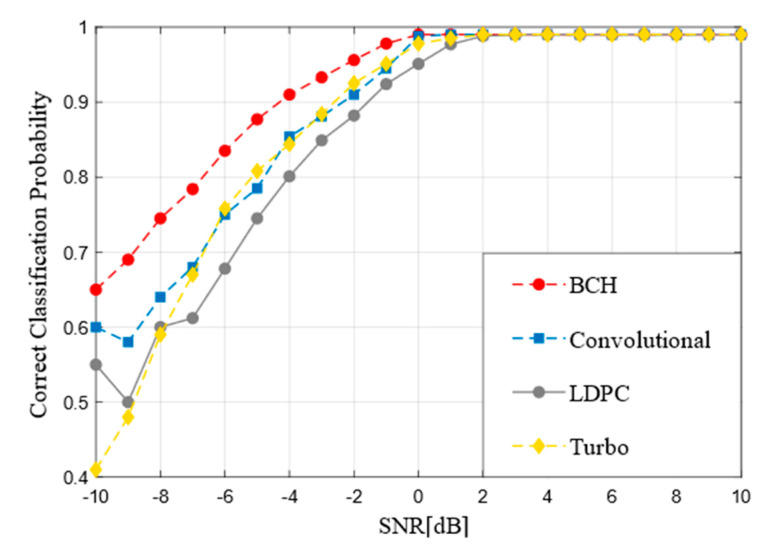
Recognition performance of the improved RNN model for each code type.

Figure 4 indicates the recognition performance of different FEC codes at −10 dB to 10 dB. It can be observed from the figure that BCH codes rise fastest, while LDPC codes and Turbo codes rise slowly from −10 dB to −5 dB. It is concluded that the more complex the coding is, the higher the SNR is needed to achieve the equivalent classification accuracy. When the SNR is greater than 0 dB, the accuracy of all coding types can reach at least 90%. With the further improvement of SNR, the accuracy of all coding types can reach 99%, all code words can almost be correctly recognized. Furthermore, with the increase of SNR, the recognition accuracy tends to be stable at 99%.

**Figure 5:**
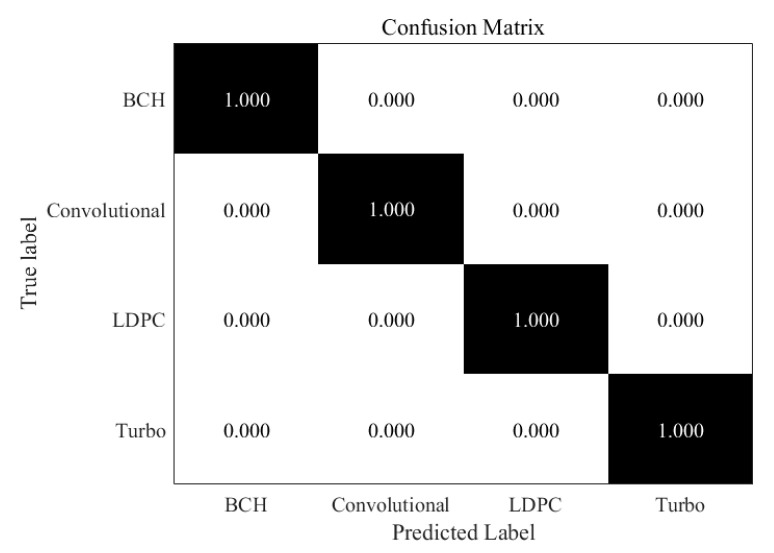
Confusion matrix for improved RNN trained at 10 dB SNR.

So as to better show the difference of the accuracy of these coding types at different SNR, we present confusion matrices for several classifiers at specific SNR and compare the differences of recognition accuracy at low (−5 dB), medium (0 dB) and high (10 dB). As indicated in the Figure 5, under the circumstance of 10 dB SNR, all code words can be recognized correctly and the classifier has good performance for various code types.

**Figure 6:**
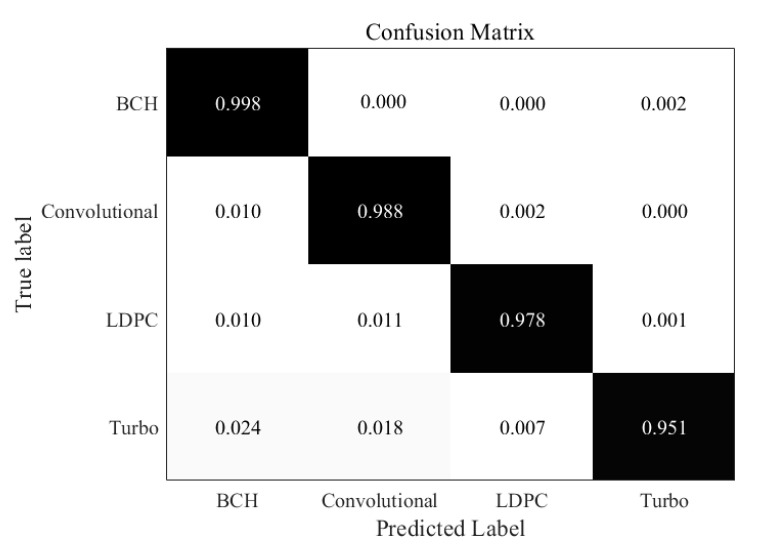
Confusion matrix for improved RNN trained at 0 dB SNR.

**Figure 7:**
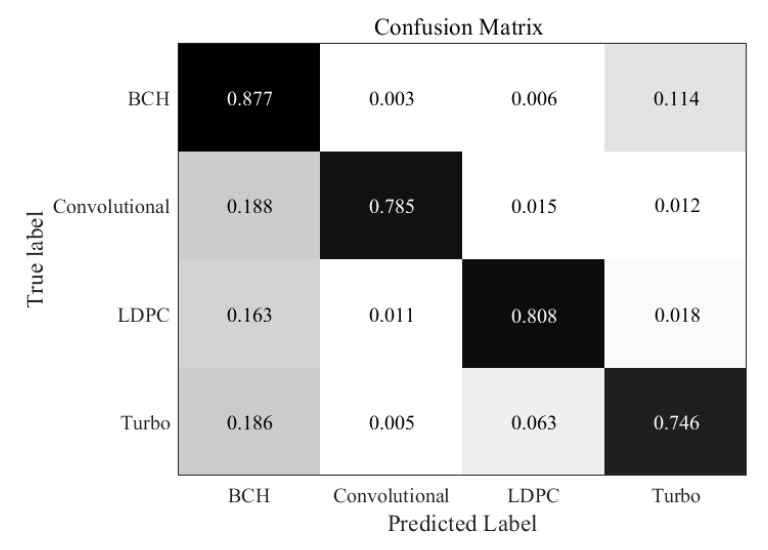
Confusion matrix for improved RNN trained at −5 dB SNR.

Similarly, in the Figure 6, when the SNR is 0 dB, errors begin to appear in all kinds of code words, but the overall recognition rate is still very high, above 95%. We can find that the recognition rate of BCH codes is the highest, because BCH code is the most basic code type, its performance is also better. Turbo code is the most misjudgment, which can be understood as a problem caused by close datasets. The confusion matrix under the condition of very low SNR is shown in the Figure 7. It shows that recognition at −5 dB SNR is challenging. However, it is still possible to recognize each type of codewords in general. The recognition performance of BCH codes is still the best, while the recognition result of Turbo codes is not ideal. Furthermore, we can also find that the convolutional codes are easier to recognize than LDPC codes and Turbo codes, this is because the context information for convolutional codes is much more remarkable than other two codes. It shows that under the condition of high noise, the recognition rate of code type of the improved RNN network still decreases obviously.

### 4.3. Performance of Input Sample Length

**Figure 8:**
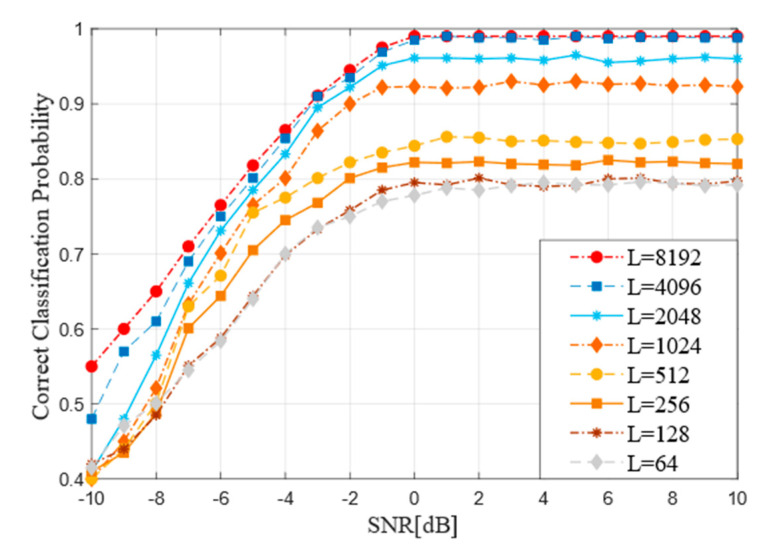
Recognition performance of the improved RNN in different lengths of input sample (L).

When other parameters of the improved RNN are not changed, the samples with the length of input samples ranging from 32 to 8192 are trained. The performance of the model is in the range of −10 dB to 10 dB. The Figure 8 indicates the influence curve of input sample length on performance.

By observing the Figure 8, under the circumstance of low signal-noise, the recognition accuracy is low regardless of the size of the sample length value and it is difficult to meet the requirements of codeword recognition. With the increase of SNR, the accuracy of network recognition performs significantly, which can meet the requirement of increasing the average accuracy by about 4% when the input length is increasing. As the sample length reaches L = 4096 or L = 8192, there is little difference in the accuracy and the overall accuracy is stable at 0.99. As the Figure 8 shows, when L is less than 2048, the recognition accuracy is significantly improved with each increase in the length level. However, when L length exceeds 4096, the performance of the model is not significantly improved. In addition, the longer the input length is, the more memory, training time and data sets are needed and the corresponding recognition efficiency will decrease.

### 4.4. Performance of Training Set Size

**Figure 9:**
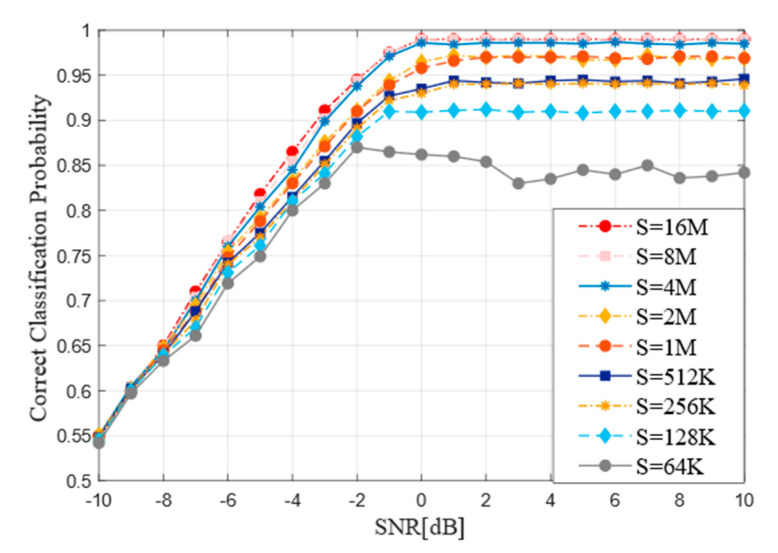
Recognition performance of the improved RNN in different sizes of training set (S).

The size of training samples is an important parameter of neural network and often has a great impact on the accuracy of network training. This experiment takes this into account formally and carries out code type recognition on nine different sizes of samples from 64K to 16M to analyze its influence. The Figure 9 shows how the curve of model performance influenced by the size of training samples. When the sample size is too low, for example, when S = 64K, the model recognition accuracy fluctuates significantly and the recognition accuracy is not high, fluctuating around 80%. With the increase of the size of samples, the identification accuracy is also increasing and the curve tends to be stable. However, when the sample size S exceeds 4M, the sample accuracy has reached 99% and the recognition rate does not fluctuate greatly with the change of SNR and no significant improvement has been observed.

### 4.5. Performance of Models Comparison

**Figure 10:**
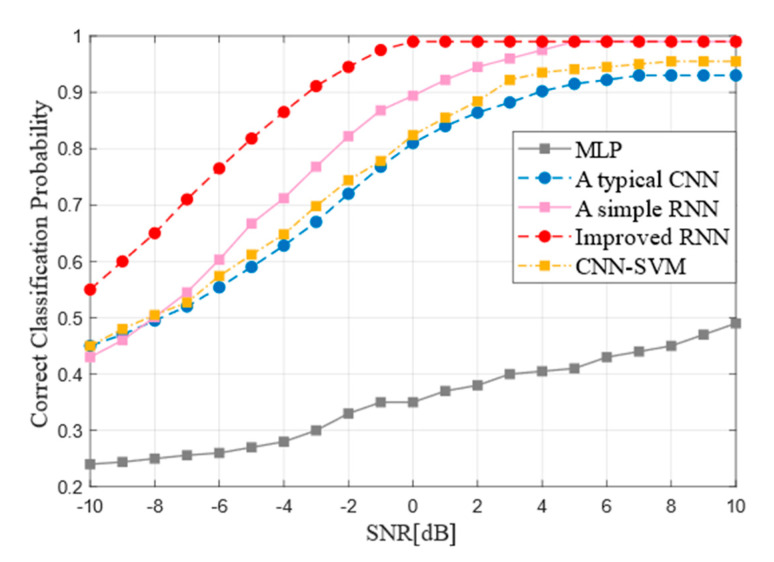
Performance comparison of the improved RNN with others.

The recognition accuracy of different deep neural network models is compared at first. the four kinds of networks are trained at the SNR of −10 dB~10 dB. The training was carried out on a data set of 8 million samples and each input sample length was 8192. The Simple RNN, the improved RNN and the typical CNN, networks performed better in the SNR of 4 dB~10 dB, as shown in the Figure 10.

When the SNR exceeds 5 dB, the recognition accuracy of Simple RNN and improved RNN can reach 99%, while that of typical CNN can reach 90%. Then, the overall recognition rates were stable. However, compared with the traditional RNN network, the improved RNN has fast convergence and large rise rate and has basically reached a high recognition rate of 99% at 0 dB. As the Figure 10 shows, regardless of the high SNR or low SNR, the overall performance of the improved RNN is superior to that of traditional CNN and Simple RNN. At the same time, the Figure 10 also summarizes the recognition performance of MLP and SVM. First proposed by Cortes and Vapnik in 1995, SVM has shown many unique advantages in solving small samples, nonlinear and high-dimensional pattern recognition and can be extended to other machine learning problems such as function fitting. SVM method is based on statistical learning theory based on VC dimension theory and structure risk minimum principle, according to the limited sample information in the complexity of the model and learning ability to seek the best compromise between, in order to get the best generalization ability. MLP is also called Artificial Neural Network (ANN), including input layer, hidden layer, output layer. Layer to layer is fully connected state. All models were compared used the same training and test data. On the condition that there is high signal-to-noise ratio, SVM can reach about 95% but MLP can just reach about 43%. The performance of the SVM model is similar to the RNN model and the latter is difficult for fully connected network to complete type recognition for forward error correction coding.

### 4.6. Complexity and Feasibility

To assess the computational complexity of each model, we calculate the average elapsed time. The simulation platform is presented as: cpu intel i7-6800K and nvidia GTX-1080 Ti GPU.

In Table 2, it’s obvious that under the same conditions, the calculation time of different neural networks is proportional to its structural complexity. MLP network only needs 1.8 s to complete the classification, but it also has the lowest classification accuracy. CNN_SVM consumes the longest time, which is 15.4 s. Considering the accuracy of it fails to reach 99%, the efficiency of CNN_SVM needs to be improved. The Improved RNN network adopted in this paper consumes 14.1 s, Compared with Simple RNN and typical CNN, the improved model requires more computation time and higher computational complexity, because first, the initial network has a deeper network structure than the typical convolutional network, second, features can be extracted without manual operation and finally, the initialization of parameters expands the feature dimension and increases the computational cost, which is acceptable in practical system and can meet the requirements of forward error correction codes type classification.

## 5. Conclusions

In the paper, we come up with a new method to settle the problem of recognition of FEC coding types in non-cooperative systems without any prior knowledge. Based on improved RNN, the method proposed can realize end-to-end feature extraction and error-correcting code type recognition. Firstly, datasets of communication signals are generated by simulating channel environment of the wireless communication system. Then, in order to improve the prediction accuracy, the performance and robustness of the proposed network are evaluated under different signal-to-noise ratios and different types of FEC codes. Finally, we compare the performance of the improved RNN in different code lengths and sizes, the results shows when the code lengths L≥1024 or sizes S≥128k, the identification accuracy will exceed 90%, which ensures saturation of accuracy and complexity in actual code type recognition. Compared with the identification accuracy and complexity, we finally conclude that improved RNN is in line with the requirements of engineering practice under the condition of non-cooperative communication and has a promising future in recognition of FEC codes.

## Figures and Tables

**Table 1:** Convolutional codes parameter descriptions.

FEC Codes	Code Rates	*n*	k	Generators
Convolutional	1/2	1	2	[23,35]
				[53,75]
				[133,171]
				[237,345]

**Table 2:** Average run time of different networks.

	Run Time(s)
Improved RNN	14.1
Simple RNN	11.4
MLP	1.8
Typical CNN	5.8
CNN_SVM	15.4

## Data Availability

Not applicable.
